# Nrf2 activation in osteoblasts suppresses osteoclastogenesis via inhibiting IL-6 expression.

**DOI:** 10.1016/j.bonr.2019.100228

**Published:** 2019-11-01

**Authors:** Tsuyoshi Narimiya, Hiroyuki Kanzaki, Yuki Yamaguchi, Satoshi Wada, Yuta Katsumata, Ken Tanaka, Hiroshi Tomonari

**Affiliations:** Department of Orthodontics, School of Dental Medicine, Tsurumi University, 2-1-3 Tsurumi, Tsurumi-ku, Yokohama, Kanagawa pref., 230-8501, Japan

**Keywords:** Nrf2, Osteoblast, Osteoclast, Osteoclastogenesis, IL-6, ALA/SFC

## Abstract

Bone destructive diseases such as periodontitis and rheumatoid arthritis are caused by excessive activation of osteoclasts. Osteoclastogenesis is regulated by Receptor activator of nuclear factor kappa-β ligand (RANKL) produced by osteoclastogenesis supporting cells such as osteoblast and osteocyte. Previously, we reported that NF-E2-related factor-2 (Nrf2) activation in osteoclast precursors inhibited osteoclastogenesis and bone destruction via induction of anti-oxidation and thereby attenuated intracellular ROS signaling. However, it still remains unknown whether Nrf2 activation in cells other than osteoclasts give any negative influence on supporting property for osteoclastogenesis. Here we discovered that Nrf2 activation in osteoblasts suppresses indirectly osteoclastogenesis via inhibiting the expression of interleukin-6 (IL-6) which promotes osteoclastogenesis. In this study, 5-aminolevulinic acid hydrochloride (ALA) and sodium ferrous citrate (SFC) was used as the Nrf2 activator. *in vitro* experiments, using osteoblast cell line, MC3T3-E1, revealed that the expression of IL-6 was increased by LPS stimulation, but decreased after ALA/SFC treatment in mRNA and protein levels. Furthermore, RANKL expression was augmented by LPS, which was blocked by ALA/SFC treatment. Neutralizing antibody against IL-6 confirmed that LPS-mediated RANKL augmentation was dependent on IL-6 induction. *in vivo* experiments with LPS-mediated bone destruction in mice, confirmed that augmented IL-6 expression in osteoblasts by immunochemical analysis. ALA/SFC treatment attenuated LPS-mediated IL-6 upregulation. These results suggest that Nrf2 activation in osteoblasts suppress IL-6 and inflammatory bone destruction. The Nrf2 activator acts not only on osteoclasts but also on osteoblasts, in other word, Nrf2 activation indirectly suppresses osteoclastogenesis. In conclusion, the Nrf2 activator exhibits dual inhibitory effects via direct action on osteoclast and indirect action on osteoclast supporting cells.

## Highlights

• ALA/SFC induced the activation of Nrf2 in osteoblasts.
• IL-6 and RANKL expression in osteoblasts was increased by LPS, but decreased by Nrf2 activation.
• LPS-mediated RANKL augmentation was dependent on IL-6 induction.
• Nrf2 activation in osteoblasts suppresses indirectly osteoclastogenesis via inhibiting the expression of IL-6.

## Graphical abstract

**Figure:**
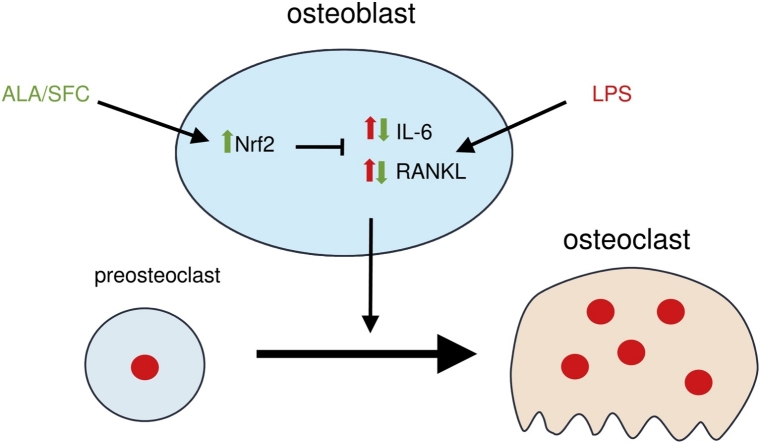

## 1 Introduction

Abnormal augmentation of osteoclastogenesis gives rise to bone destruction in diseases such as periodontitis (Fujikawa et al., 1996). It is well known that the receptor activator-kappaB ligand (RANKL)/RANK system controls osteoclastogenesis (Teitelbaum and Ross, 2003). RANKL is expressed by supporting cells such as osteoblasts and fibroblasts (Boyle et al., 2003). In the bone destructive stage of rheumatoid arthritis, it has been reported that expression of RANKL in synovial fibroblasts is increased, thereby causes bone destruction via promoting osteoclastogenesis (Danks et al., 2016). It is known that ROS is a signal molecule downstream of RANK (Bax et al., 1992; Ha et al., 2004; Kanzaki et al., 2014), and the reduction of ROS inhibits osteoclastogenesis and osteoclast activation (Li et al., 2018).

Oxidative stress such as ROS exhibits cytotoxicity against cells (Wells et al., 2009), therefore cell have protective mechanisms against these oxidative stressors (Furukawa-Hibi et al., 2005; Kensler et al., 2007). Among these protective mechanisms, Nrf2 is a transcription factor that regulates anti-oxidative enzymes and protects cells from oxidative stress (Kobayashi et al., 2016). We previously reported that the activation of Nrf2 in osteoclasts suppresses osteoclastogenesis and bone destruction (Kanzaki et al., 2013). Nrf2 activation causes a rise in cytoprotective enzymes, which decreases intracellular ROS.

It has been reported that inflammatory cytokines such as IL-1β, IL-6 and TNF-α express in rheumatoid arthritis (RA) and induces osteoclastogenesis via increasing the expression of RANKL (Pi et al., 2003; Mori et al., 2011). IL-6 is a pro-inflammatory cytokine secreted from the cells such as fibroblasts and osteoblasts (Zhang et al., 1988; Ishimi et al., 1990), and is upregulated in inflammatory lesions such as RA (Braun and Zwerina, 2011). More importantly, it has been reported that IL-6 promotes osteoclastogenesis via upregulating the expression of RANKL (Ishimi et al., 1990; Mihara et al., 2012).

We have previously reported that the activation of Nrf2 in osteoclasts suppresses osteoclastogenesis (Kanzaki et al., 2013). However, it still remains unknown whether the activation of Nrf2 in osteoblasts inhibits osteoclastogenesis supporting activity. In this study, we clarify the activation of Nrf2 in osteoblasts attenuates on inflammatory cytokine production, and thereby indirectly inhibits osteoclastogenesis.

## 2 Materials and methods

### 2.1 Chemicals

ALA were purchased from Wako Pure Chemical (Osaka, Japan). SFC was a gift from Eisai Food and Chemical (Tokyo, Japan). Purified LPS from Escherichia coli 0111: B4 (Sigma-Aldrich, St. Louis, MO, USA) was dissolved in PBS at concentration of 1 mg/ml. Brefeldin A Solution was purchased from Biolegend (San Diego, CA).

### 2.2 Cells

The MC3T3-E1 mouse calvaria-derived cell line was obtained from RIKEN BioResource Research Center (Tsukuba, Japan).

### 2.3 Cell culture

MC3T3-E1 Cells were cultured in α-modified Eagle’s medium (Wako-Pure Chemical, Osaka, Japan) that contained 10％ fetal bovine serum (Thermos Scientific, South Logan, UT) supplemented with antibiotics (100 U/mL of penicillin and 100 μg/mL of streptomycin). They were cultured at 37 °C in a 5% CO_2_ incubator.

### 2.4 Real-time RT-PCR analysis

RNA was extracted from MC3T3-E1 Cells using NucleoSpin® RNA (Macherey-Nagel, Düren, Germany) with on-column genomic DNA digestion according to the manufacturer’s instructions. RNA of MC3T3-E1 cells were extracted after treatment of cells with or without 1.0 μg/ml LPS for 6 h, 24 h, 48 h and 72 h. In order to investigate effects of ALA and SFC, RNA were extracted after cultivation with or without ALA and SFC at 6 h and 24 h. To investigate effects of IL-6 for RANKL expression in osteoblasts, MC3T3-E1 was pretreated with monoclonal rat anti-mouse anti-IL-6, neutralizing antibody (0.5 μg/ml; Biolegend, San Diego, CA) for 30 min and treated with LPS. RNA were extracted after cultivation with or without anti-IL-6. After measurement of the RNA concentration, isolated RNA (500 ng each) was reverse transcribed with iScript cDNA-Supermix (Bio-Rad Laboratories, Hercules, CA), and cDNA was diluted (10×) with Tris-EDTA buffer. Real-time RT-PCR was performed with SsoFast EvaGreen-Supermix (Bio-Rad Laboratories). Primer sequences used for the experiments were as follows: mouse Rps18: (F) 5'-AGTTCCAGCACATTTTGCGAG-3' and (R) 5'-TCATCCTCCGTGAGTTTCTCCA-3', mouse Nrf2: (F) 5'-GCCCACATTCCCAAACAAGAT-3' and (R) 5'-CCAGAGAGCTATTGAGGGACTG-3', HO-1: (F) 5'-AAGCCGAGAATGCTGA-3' and (R) 5'-GCCGTGTAGATATGGTACAAGGA-3', mouse IL-6: (F) 5'-TAGTCCTTCCTACCCCAATTTCC-3', (R) 5'-TTGGTCCTTAGCCACTCCTTC-3', mouse RANKL: (F) 5'-CAGCATCGCTCTGTTCCTGTA-3' and (R) 5'-CTGCGTTTTCATGGAGTCTCA-3'. Fold changes of gene of interest were calculated with ΔΔCt method using Rps18 as a reference gene.

### 2.5 Western blot analysis for IL-6

MC3T3-E1 was treated with ALA and SFC for 1 h and then treated with LPS for 24 h. Brefeldin (3.0 μg/ml) were used to inhibit protein transport during culture. Cells were washed with PBS and treated with cell lysis buffer (5 mM EDTA, 10％ glycerol, 1％ Triton X-100, 0.1% SDS, 1％ NP-40) in PBS. Protein concentration in each of the lysates was measured with Pierce BCA Protein Assay kit (Thermo Fisher Scientific, Waltham, MA) and adjusted to be the same for each lysate. After mixing with sample buffer, it was heat denatured, were electrophoresed on a TGX Precast gel (Bio-Rad Laboratories). The proteins were transferred to a PVDF membrane, and blocked with PVDF Blocking Reagent (Toyobo Co. Ltd, Osaka, Japan). Membrane was then incubated with anti-IL6 antibody (1/2000 dilution; ProteinTech Group, Chicago, IL, USA). After washing 0.5% Tween-20 in PBS (PBS-T), the membrane was incubated with HRP-conjugated secondary antibody (Thermo Fishter Scientific, San Jose, CA). To confirm the amount of the loaded protein were equal, membrane was incubated with anti-β Actin antibody (FUJIFILM Wako Pure Chemical Corporation, Osaka, Japan). Chemiluminescence was produced using Luminata Forte (EMD Millipore Corporation, Billerica, MA) and detected with LumiCube (Liponics, Tokyo, Japan).

### 2.6 Measurement of interleukin-6 in the culture supernatant

MC3T3-E1 was treated with ALA and SFC for 1 h, then treated with LPS for 24 h and then the supernatant was collected. Concentration of IL-6 in the culture supernatant of MC3T3-E1 cells was measured using a commercially available mouse IL-6 ELISA development kit (Biolegend, San Diego, CA). All samples were measured with ×20 dilution by PBS in triplicate.

### 2.7 Animals and experimental bone destruction

All experimental protocols were approved by the Institutional Animal Care and Use committee, Tsurumi University (approval numbers; 26A081). All animals were treated ethically, and animal experiments were carried out in accordance with the Guidelines for Animal Experimentation of Tsurumi University.

We utilized repeat injections of LPS for *in vivo* bone destruction model (Kanzaki et al., 2014; Ayon Haro et al., 2011; Kanzaki et al., 2017). Bone destruction in mice calvaria was induced with injection of LPS (10 μg/site). The five injections were performed at a point on the midline of the skull located between the ears and the eyes on every other day. Twenty 7-wk-old BALB/c male mice (Clea Japan, Tokyo, Japan) were used in experiments. They were divided into the following 4 groups (n = 5 each). G1 (control group); a PBS-injected group, G2 (SFC/ALA group); an SFC/ALA-injected group, G3 (LPS group); LPS-induced bone resorption group; G4 (LPS + SFC/ALA group); an LPS-injected bone resorption and SFC/ALA-injected group. On 24 h after final injection, mice were euthanized by cervical dislocation and cranial tissue samples were fixed overnight with 4％ paraformaldehyde in PBS.

### 2.8 Preparation of paraffin sections

After washing with PBS, fixed cranial tissue samples were decalcified with 10％ EDTA in PBS, dehydrated, and embedded in paraffin. The specimens were examined in serial coronal Sections (6 μm-thick).

### 2.9 Immunohistochemical analysis

After deparaffinization, the sections were incubated with 0.3％ H_2_O_2_ in methanol to quench the endogenous peroxidase activity and then treated with BLOCK ACE (DS PHARMA BIOMEDICAL, Osaka, Japan). The sections were incubated with anti-IL-6 antibody (ProteinTech Group). After washing with PBST, the sections were incubated with peroxidase-conjugated secondary antibody (Vector Laboratories, Burlingame, CA). After incubation, the sections were flooded with DAB solution (Vector Laboratories), counterstained with hematoxylin. Sections were mounted with Entellan (Merck) and observed with a microscope. Intensity levels were measured using Image J (colour deconvolution) (Schneider et al., 2012).

### 2.10 Statistical analysis

All data are presented as the mean ± standard deviation from three independent experiments. ANOVA and Tukey’s HSD test were used for evaluating the statistical significance (SPSS® 11.0 J; IBM, Chicago, IL). P < 0.05 was considered statistically significant.

## 3 Results

### 3.1 ALA/SFC activates Nrf2-mediated anti-oxidation in osteoblasts

**Fig. 1:**
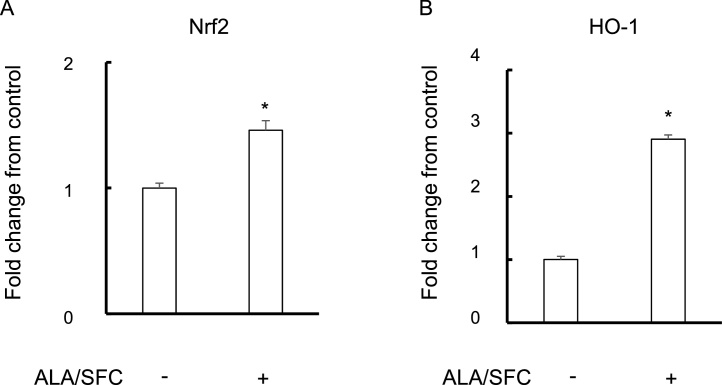
ALA/SFC activates Nrf2-mediated HO-1 in osteoblasts. Real-time RT-PCR analysis for Nrf2 (A) and HO-1 (B) expression at 48 h. Fold change from control is shown. * P < 0.05 versus control.

We firstly examined whether the ALA/SFC activated the expression of Nrf2. Real-time PCR analysis revealed that the expression of Nrf2 in MC3T3-E1 cells was increased by ALA/SFC (Fig. 1A). Several studies found that ALA/SFC mediated the induction of HO-1 via the activation of Nrf2 (Nishio et al., 2014; Fujino et al., 2016). Consistently, real-time PCR analysis revealed that the expression of HO-1 in MC3T3-E1 cells was increased by ALA/SFC (Fig. 1B). These results indicated that ALA/SFC effectively activates Nrf2-mediated anti-oxidation in osteoblasts.

### 3.2 IL-6 expression was induced in osteoblasts by LPS stimulation

**Fig. 2:**
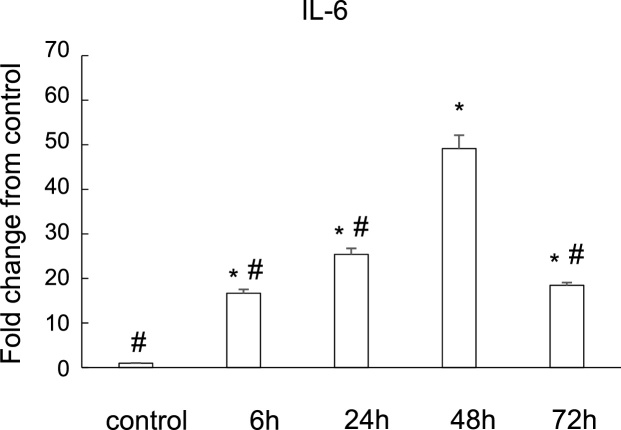
IL-6 expression was induced in osteoblasts by LPS. Real-time RT-PCR analysis for IL-6 expression at 0, 6, 24, 48, and 72 h. Comparison of different time point after LPS treatment are shown. Fold change from control (0 h) is shown. * P < 0.05 versus control; # P < 0.05 versus 48 h.

We then examined whether MC3T3-E1 promoted the expression of IL-6, inflammatory cytokine, with LPS (Fig. 2). Real-time PCR analysis revealed that the expression of IL-6 in MC3T3-E1 cells was increased in a time-dependent manner for up to 48 h but the expression was lower at 72 h. These results suggest that inflammatory stimulation favors osteoclastogenesis with induction of osteoclastogenic cytokine, IL-6.

### 3.3 The Nrf2 activation of osteoblasts suppressed expression of IL-6 at the mRNA level

**Fig. 3:**
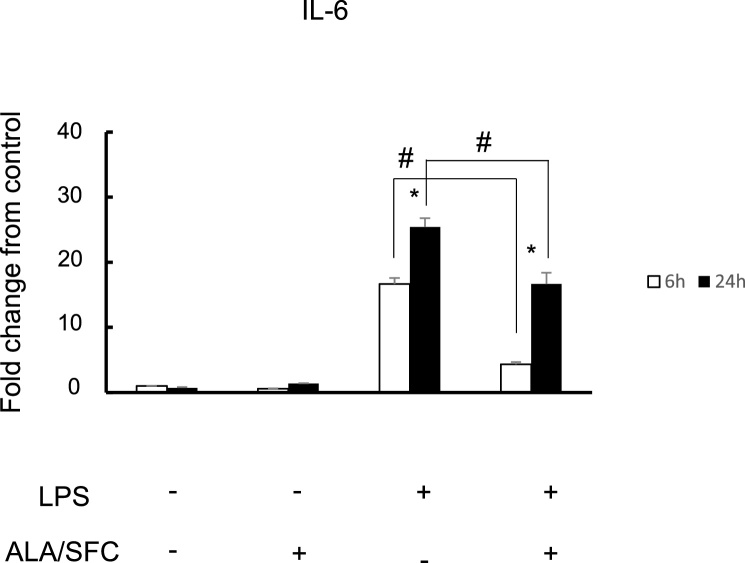
Nrf2 activation in osteoblasts suppresses IL-6 expression at the mRNA level. Real-time RT-PCR analysis for IL-6 expression at 6 and 24 h. Fold change from control is shown. * P < 0.05 versus control; # P < 0.05 between the samples. open bar: 6 h close bar: 24 h.

We then examined whether the activation of Nrf2 in MC3T3-E1 cells suppressed IL-6 expression. The expression of IL-6 in the cells stimulated by LPS was significantly decreased by ALA/SFC at 6 h and 24 h (Fig. 3). These results suggest that Nrf2 activation downregulates IL-6 expression induced by LPS.

### 3.4 The Nrf2 activation of osteoblasts suppressed the expression of IL-6 at the protein level

**Fig. 4:**
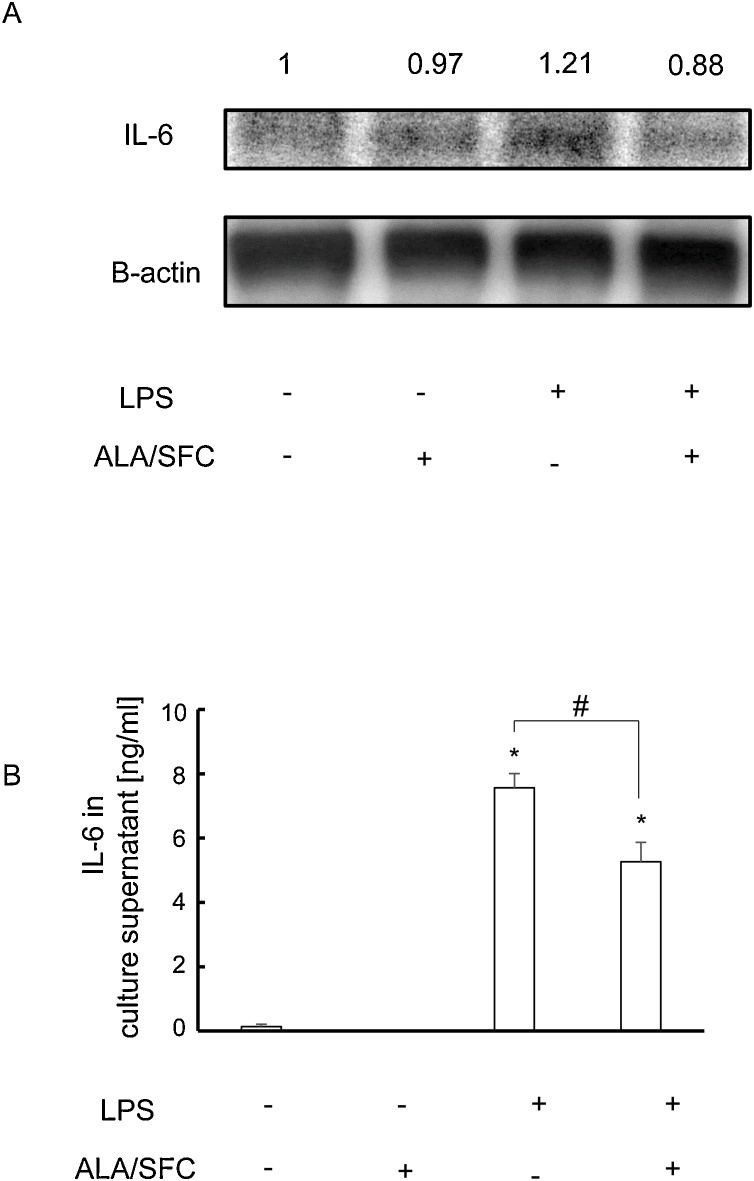
Nrf2 activation in osteoblasts suppresses the expression of IL-6 at the protein level. (A)Western blot analysis of IL-6 and β-actin using cell lysates at 24 h. Representative images of IL-6 (top panel) and β-actin (bottom panel) are shown. Fold change from control are shown above. (B) IL-6 concentration in the culture supernatants measured by ELISA. *: P < 0.05 versus control; # P < 0.05 between the samples.

We further examined protein level expression of IL-6 by western blot analysis and ELISA whether the activation of Nrf2 in MC3T3-E1 cells suppressed the expression of IL-6 (Fig. 4 A and B). In the comparison between the control and the LPS stimulated samples by western blot analysis, a dense band was observed in the LPS stimulated samples (Fig. 4A). In the comparison between the LPS stimulated and the LPS + ALA/SFC samples, thin band was observed in the LPS + ALA/SFC samples. Consistently, ELISA revealed the reduced IL-6 production in LPS + ALA/SFC samples than in LPS stimulated samples (Fig. 4B). These results indicated that the activation of Nrf2 activation with ALA/SFC suppressed IL-6 even at protein level.

### 3.5 The Nrf2 activation in osteoblasts suppresses RANKL

**Fig. 5:**
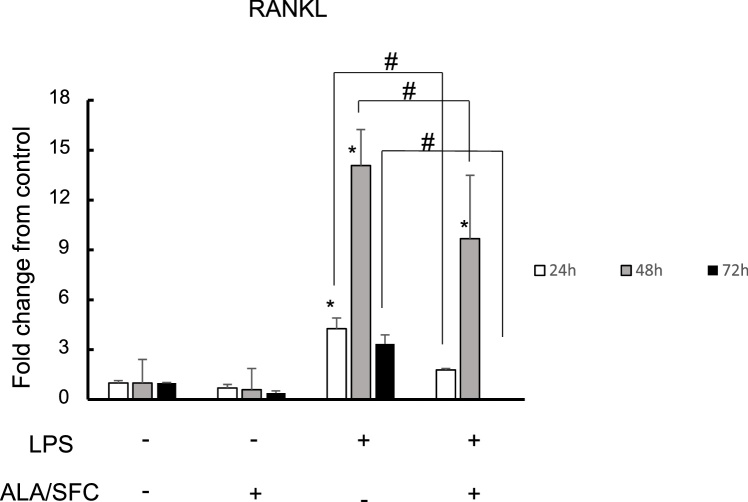
Nrf2 activation in osteoblasts suppresses RANKL expression at the mRNA level. Fold change from control at each time point are shown. *: P < 0.05 versus control; # P < 0.05 between the samples. open bar: 24 h gray bar: 48 h closed bar: 72 h.

Next we examined whether the activation of Nrf2 suppressed RANKL expression (Fig. 5). The expression of RANKL was increased by LPS and it was peak at 24 h. This LPS-mediated induction of RANKL was suppressed by ALA/SFC. These results suggest that Nrf2 activation downregulates RANKL expression induced by LPS.

### 3.6 Anti-IL-6 antibody suppressed RANKL expression induced by LPS in osteoblasts

**Fig. 6:**
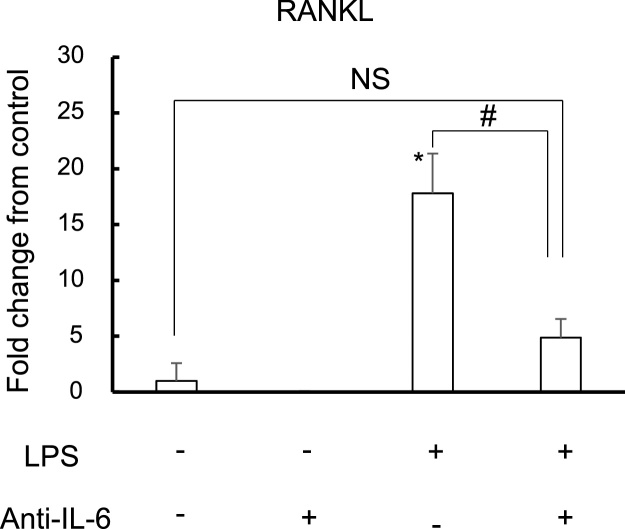
Anti-IL-6 antibody suppresses RANKL expression in osteoblasts induced by LPS. Real-time RT-PCR analysis for RANKL expression at 48 h. Fold change from control is shown. *: P < 0.05 versus control; # P < 0.05 between the samples. NS: not significant difference between the samples.

We examined whether the anti-IL-6 neutralizing antibody suppressed RANKL expression induced by LPS in osteoblasts. RANKL expression induced by LPS was significantly decreased by anti-IL-6 antibody (Fig. 6). These results suggest that IL-6 promotes osteoclastogenesis on augmentation of RANKL expression in osteoblasts.

### 3.7 Local Nrf2 activation attenuated LPS-mediated IL-6 augmentation in vivo

**Fig. 7:**
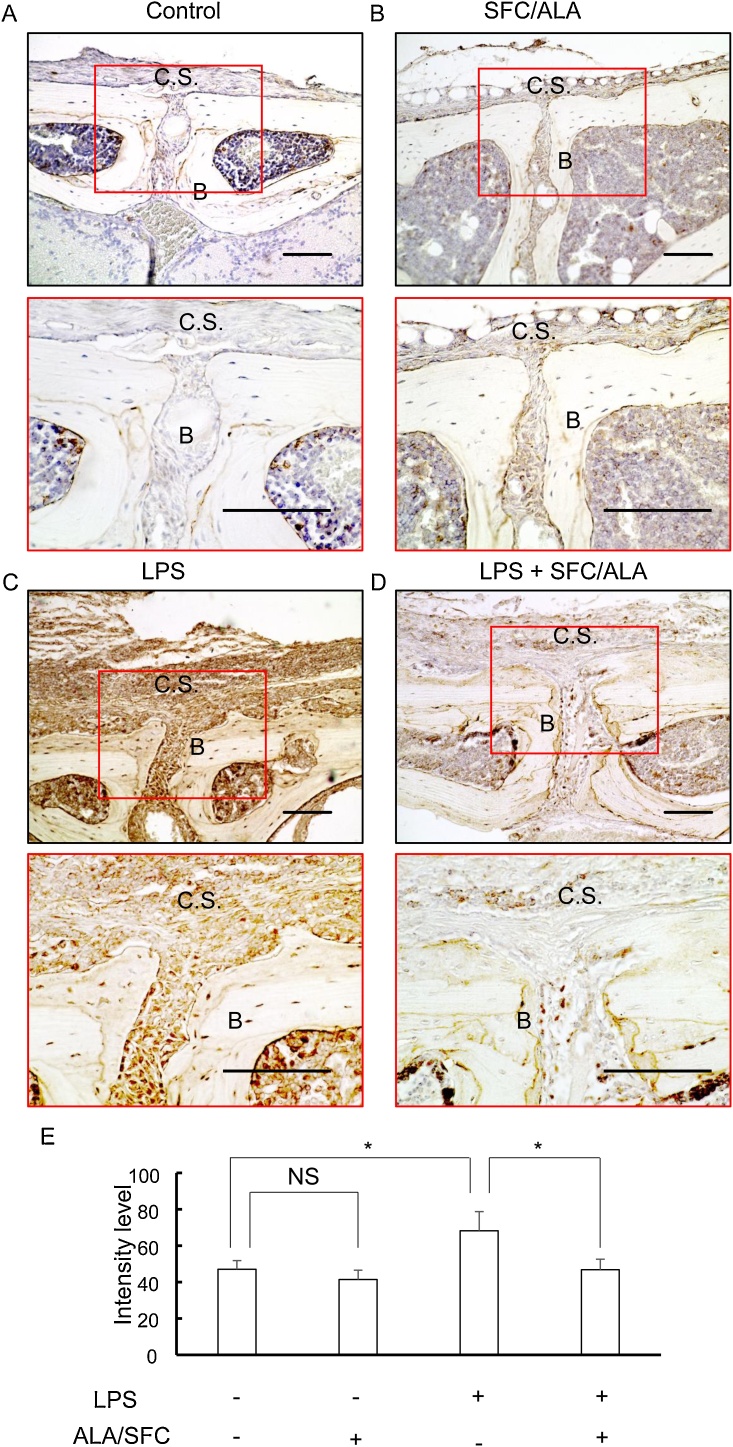
Local ALA/SFC injection ameliorates LPS-induced IL-6 production in mice. (A–D) Immunohistochemical staining for IL-6 (n = 5). Representative images of control without LPS nor ALA/SFC (A), experiment group with ALA/SFC without LPS, (B) experiment group with LPS without ALA/SFC (C), and experiment group of with LPS and ALA/SFC (D) are shown. The image of each experiment group at lower magnification (the upper panel) and higher magnification (the lower panel) are shown. C.S.: calvaria surface B: bone Bar = 100 μm. (E) The intensity of the immuno-reactivity for IL-6. Mean value of 3 sections are shown. *: p < 0.05 versus control.

To further investigate IL-6 expression in the bone destruction model, immunohistochemistry was performed (Fig. 7). Compared with control group, there was no significant difference in the expression of IL-6 in ALA/SFC group. In LPS group, IL-6 was extensively expressed in cells including osteoblasts on the outer surface layer of the calvariae. Surprisingly the expression of IL-6 was markedly reduced in LPS + SFC/ALA group. These results suggest that Nrf2 activation with ALA/SFC suppresses the IL-6 production induced by LPS.

## 4 Discussion

In the research, we clarified that Nrf2 activation in osteoclastogenesis supporting cells attenuated IL-6 production, and thereby inhibited osteoclastogenesis. Since we have previously been reported that Nrf2 activation in osteoclast precursors directly inhibit osteoclastogenesis, our present report shed light on the indirect inhibitory effect of Nrf2 activation on osteoclastogenesis via attenuation of inflammatory cytokine production.

It is important to suppress bone destruction for the treatment of bone destructive diseases such as periodontitis and rheumatoid arthritis (Belibasakis and Bostanci, 2012; Hirano et al., 1988). Our previous studies have shown that Nrf2 activation of osteoclasts suppresses RANKL-dependent osteoclastogenesis through suppression of oxidase stress (ROS) signaling (Kanzaki et al., 2014, 2013; Kanzaki et al., 2017, 2015). However, it was not clear whether Nrf2 activation in cells other than osteoclasts affects osteoclastogenesis. In this study, we focused on osteoblasts, which are important for bone remodeling (Tanaka et al., 2005). We used 5-aminolevulinic acid hydrochloride (ALA) and sodium ferrous citrate (SFC) as Nrf2 activators and examined the effect on osteoclastogenesis by the activation of Nrf2 in osteoblasts (Nishio et al., 2014). The activation of Nrf2 suppresses the secretion of IL-6 in osteoblasts, and indirectly suppresses osteoclastogenesis by the reduction of IL-6 which promotes osteoclastogenesis.

It is known that inflammatory cytokines as IL-1β, TNF-α and IL-6 increase in bone destructive diseases (Ishimi et al., 1990; Kobayashi et al., 1994; Chaabo and Kirkham, 2015). These cytokines increase the expression of RANKL, and promotes osteoclastogenesis (Mori et al., 2011). In this study, it was confirmed that IL-6 was involved in the upregulation of RANKL induced by LPS in osteoblasts using anti-IL-6, neutralizing antibody.

Essentially, the functions of osteoblasts and osteoclasts are antagonistic (Tanaka et al., 2005). When the balance between osteoclasts and osteoblasts activity collapses, bone resorption occurs. In this study, LPS was used to mimic inflammatory bone destructive diseases where pro-inflammatory cytokines were increased and promoted osteoclastogenesis.

Innate immune responses triggered by LPS are mediated by Toll-like receptor 4 (TLR4) (Hoshino et al., 1999). LPS engagement via the TLR4 receptor complex triggers signaling pathway. The signaling pathways is dependent on the myeloid differentiation primary response gene 88 (myD88) protein (Hoshino et al., 1999). The MyD88-dependent pathway involves recruitment of tumor necrosis factor (TNF)-receptor associated factor 6 (TRAF6), which results in activation of TGF**β**-activated kinase 1 (TAK1), and leading to activation of NF-κB. NF-κB translocates to the nucleus to the nucleus to promote the transcription of IL-6 gene. Nrf2 was reported as a key transcription factor that interacts with NF-κB (Wardyn et al., 2015). We presumed that Nrf2 activation transcriptionally attenuates NF-κB-dependent inflammatory cytokine expression such as IL-6 and RANKL.

Our data clearly demonstrated that Nrf2 activation attenuated LPS-mediated cytokine induction. It is presumed that activation of Nrf2 removes the oxidative stress by enhanced anti-oxidative stress enzymes such as HO-1 and GCS, and indirectly suppresses inflammation (Pi et al., 2003). In addition, it has been reported that Nrf2 in macrophages binds to upstream of IL-6 gene and negatively regulates cytokine production (Kobayashi et al., 2016). Together, it was inferred in this study that IL-6 gene in the osteoblasts was suppressed with Nrf2. Further exploration is necessary to clarify the negative regulatory mechanism of inflammatory cytokine production by Nrf2.

In conclusion, we discovered that Nrf2 activator exhibits dual inhibitory effects via direct action on osteoclast and indirect action on osteoclast supporting cells. This suggests that the Nrf2 activator can be used for an effective therapeutic agent against inflammatory bone destructive diseases such as periodontitis and rheumatoid arthritis.

## Declaration of Competing Interest

None.
